# Knowledge Mining from Clinical Datasets Using Rough Sets and Backpropagation Neural Network

**DOI:** 10.1155/2015/460189

**Published:** 2015-03-04

**Authors:** Kindie Biredagn Nahato, Khanna Nehemiah Harichandran, Kannan Arputharaj

**Affiliations:** ^1^Ramanujan Computing Centre, Anna University, Chennai, Tamil Nadu 600025, India; ^2^Department of Information Science and Technology, Anna University, Chennai, Tamil Nadu 600025, India

## Abstract

The availability of clinical datasets and knowledge mining methodologies encourages the researchers to pursue research in extracting knowledge from clinical datasets. Different data mining techniques have been used for mining rules, and mathematical models have been developed to assist the clinician in decision making. The objective of this research is to build a classifier that will predict the presence or absence of a disease by learning from the minimal set of attributes that has been extracted from the clinical dataset. In this work rough set indiscernibility relation method with backpropagation neural network (RS-BPNN) is used. This work has two stages. The first stage is handling of missing values to obtain a smooth data set and selection of appropriate attributes from the clinical dataset by indiscernibility relation method. The second stage is classification using backpropagation neural network on the selected reducts of the dataset. The classifier has been tested with hepatitis, Wisconsin breast cancer, and Statlog heart disease datasets obtained from the University of California at Irvine (UCI) machine learning repository. The accuracy obtained from the proposed method is 97.3%, 98.6%, and 90.4% for hepatitis, breast cancer, and heart disease, respectively. The proposed system provides an effective classification model for clinical datasets.

## 1. Introduction

In this information era the advancement of computerized database system facilitates the enhancement of decision making and diagnosis in medical science. Analysis of clinical datasets by using data mining methodology, techniques, and tools helps to develop a knowledge based system that can assist clinicians in decision making [1, 2]. Clinical dataset consists of information about the current condition of the patients [3]. The information includes data of patient profile, physical checkup, and laboratory results. Mining knowledge from clinical dataset refers to the discovery of hidden valuable knowledge, to develop clinical expert system [3, 4].

Knowledge mining methodology applied on the clinical dataset depends on the type of dataset and the purpose to attain the desired objective. The major data mining functionalities are association rule mining, classification, and clustering [1]. Association rule discovers interesting relationship among the attributes. The interestingness of the relation is measured using two metrics, namely, support and confidence [1, 5]. Classification is applied on a dataset which has a predefined class. Classification process learns from a set of attributes that include decision attribute to decide the class label. The learning process is done by a learning algorithm applied over a training dataset, whereas the performance of learning is evaluated using performance measures over the testing dataset [2]. Clustering is performed on a dataset to categorize it into a group by maximizing the similarity and minimizing the difference in a group. The learning technique used in classification is supervised whereas in clustering it is unsupervised [1, 6].

In this work a classifier that predicts the presence or absence of a disease using rough set indiscernibility relation method and backpropagation learning algorithm is developed.

Rough set theory, proposed by Pawlak during 1980s, deals with uncertainty, vagueness, imprecision, and incomplete information [7–9] for feature selection, feature reduction, and extraction of decision rule from the given dataset. Indiscernibility relation method of rough set theory is used to select minimal representative subset of the attributes from the dataset. The selected attributes are used as inputs to the backpropagation neural network learning process. Backpropagation (BP) method is commonly used in artificial neural network (ANN) model proposed by Rumelharth [10, 11]. Backpropagation neural network (BPNN) consists of three types of layers, namely, input layer, one or more hidden layers, and output layer [11, 12]. BPNN has to perform three tasks, namely, feedforward from input layer to output layer, compute and backpropagate the error, and update the weights from output layer to input layer [10, 12].

The proposed technique has been applied on three different clinical datasets obtained from University of California at Irvine (UCI) machine learning repository. These datasets are hepatitis, Wisconsin breast cancer, and Statlog heart disease dataset [13].

Hepatitis refers to inflammation of the largest internal human organ, liver, caused by viral infection. The hepatitis virus can be grouped as A, B, C, D, and E. These viruses cause acute to chronic disease; type B and type C, especially, lead to chronic diseases.

Cancer is a major cause for high mortality globally. According to World Health Organization (WHO) statistics, half a billion women died in 2011 due to breast cancer. The uncontrolled growth of cells creates mass of tissue called tumor. The tumor can be categorized as benign (not cancerous) and malignant (cancer). Breast cancer caused by a malignant tumor develops in the cells of breast.

Cardiovascular disease (CVD), which affects the heart, blood vessels, and circulation of blood throughout the body, is one of the leading causes of death. As stated by WHO, about 17 million people (30% of global death) died in 2008 due to CVD and this number will increase to 23 million in the next one and half decades.

The rest of the paper is organized as follows.  Section 2 explains the related work carried out by other researchers and highlights the significance of this work. System architecture of the proposed work is discussed in Section 3. Experimental results are discussed in Section 4. Conclusion and scope for future work are mentioned in Section 5.

## 2. Literature Review

Shao et al. [14] in their work have proposed a hybrid intelligent modeling system for heart disease classification. The dataset used by the researchers consists of 899 patients' records with 14 attributes. They have used instance elimination method for handling missing values. After removal of records with missing values, the dataset reduces to 280 records. The researchers have used single stage modeling, namely, logistic regression (LR), multivariate adaptive regression splines (MARS), rough sets (RS), and artificial neural network (ANN). The researchers have applied backpropagation network (BPNN) for ANN structure. The hybrid intelligent models used in their work are LR-ANN, MARS-ANN, RS-ANN, LR-MARS, RS-MARS, LR-RS, MARS-RS, MARS-LR, and RS-LR. The researchers have achieved an accuracy of 83.93% for the MARS-LR hybrid intelligent model with six attributes. It can be inferred from their work that the accuracy of hybrid models is higher than single stage models.

Kaya and Uyar [15] in their work developed hybrid decision support system based on rough set and extreme learning machine for diagnosis of hepatitis disease. They have used hepatitis disease dataset from UCI repository. The authors produce 20 reducts with a range of three to seven attributes using rough set. The reducts are selected before removing the missing value. The records with missing value are removed from each reduct. Classification is done by means of extreme learning machine. The researchers have obtained 100% accuracy with the reduct of four attributes (fatigue, malaise, protime, and histology) with the division of 80–20% for training-testing. The selected reduct holds only 87 records.

Sartakhti et al. [16] have designed a hybrid system of support vector machine and simulated annealing (SVM-SA) for detecting hepatitis disease. The researchers have taken the dataset from UCI repository. By removing the missing value, the researchers have reduced the size of the dataset to 80 samples. The class label “Die” has 67 samples and the remaining 13 samples are from the class “Live.” To avoid the numerical differences, the researchers have normalized the dataset. The SVM-SA method is applied with 10-fold cross validation procedure and tuned parameters are used for enhancing the classification accuracy. They achieved accuracy of 96.25%.

By using weighted fuzzy rule, Anooj [17] has developed heart disease risk prediction technique. The researcher has used three heart disease datasets from the UCI repository, namely, Cleveland data, Hungarian data, and Switzerland data. The researcher has applied preprocessing stage for removing missing values and other noisy information from the selected dataset. The researcher has grouped the instances based on the class label and then changed numerical data type to categorical based on equi-width technique. After discretization, the researcher has selected attributes for the fuzzy rule base, based on the occurrence of the attribute value in each class. The weighted fuzzy rule is applied to Mamdani fuzzy inference system. Accuracy (training-testing) of the generated IF-THEN rule has become 0.51–0.62, 0.71–0.47, and 0.36–0.51 for Cleveland, Hungarian, and Switzerland dataset, respectively.

Chen et al. [18] have built automatic diagnostic system by using support vector machine (SVM) with rough set based feature selection. It is tested using Wisconsin breast cancer dataset. The researcher discarded 16 records due to their missing value. After removal of missing value, the dataset consists of 444 samples belonging to benign class and 239 samples to malignant class. They produce 20 reducts by using genetic algorithm based rough set approach. Seven reducts have been selected for further classification process. Before classification, the researchers have normalized the dataset to the range of [−1,1]. They have performed 5-fold cross validation with the data division of 80%, 70%, and 50% training datasets. The researchers have fine-tuned the parameter of SVM to obtain higher accuracy. With the reduct of five attributes, the researchers have achieved 100% accuracy.

Marcano-Cedeño et al. [19] proposed breast cancer classification method by using artificial metaplasticity multilayer perceptron (AMMLP). The researchers have used UCI Wisconsin breast cancer dataset. By removing the sample with missing value, the remaining 683 samples are taken for further analysis. Their proposed network is trained by 60% of the dataset, that is, 410 samples. The remaining 273 samples are used for testing. Their proposed AMMLP has a single hidden layer with eight neurons. They used two criteria to stop the training process. The first one is if the mean squared error is approached to 0.01 and the other one is by fixing the training epochs to 2000. Their classifier achieves accuracy of 99.26%.

Chen et al. [20] applied a hybrid of local Fisher discriminant analysis (LFDA) and support vector machines (SVM) for hepatitis disease prediction. The proposed system was implemented on hepatitis dataset from UCI. The researchers imputed all the missing values and then normalized the dataset between an interval of [0,1]. They have reduced the dimension of the data from 19 to 2 components by using LFDA. The researchers distribute the dataset to training-testing partition of (80–20%, 70–30%, and 50-50%) by stratified sampling techniques. They have used the SVM classifier with 10-fold cross validation. The researchers fine-tuned the two parameters of SVM: regularization (*C*) and kernel function parameter (*γ*) to 0.5 and 2, respectively. They have achieved 96.77% accuracy with the data division of 80–20% for training-testing. In their study, the researchers have imputed the attribute with more than 40% of missing value attributes.

Çalişir and Dogantekin [21] proposed an intelligent system using principal component analysis (PCA) and least square support vector machine (LSSVM). The proposed method is applied on hepatitis dataset from UCI. The researchers have reduced the dimension to 10 using PCA. Their classifier, LSSVM, includes two parameters: the width of Gaussian kernel (*σ*) and regularization factor (*C*). They have obtained accuracy of 96.12% with a fine-tuned value of 0.8 and 100 for *σ* and *C*, respectively. Their proposed method did not explain whether the missing value is rejected or imputed.

Vijaya et al. [22] developed an intelligent model by using fuzzy neurogenetic approach for predicting the severity of cardiovascular disease. Their proposed method was tested on Cleveland heart disease dataset from UCI repository. The researchers have used trapezoidal membership function for fuzzification process. Their architecture holds 35, 13, and 5 neurons in the input layer, hidden layer, and output layer, respectively. Sigmoidal activation function has been applied for both hidden layer and output layer neurons. They have selected the weight associated between nodes based on the best fitness value of the chromosome by using genetic algorithm. Their system produced IF…THEN rules with an accuracy of 88%.

Dogantekin et al. [23] have proposed diagnosis system based on linear discriminant analysis and adaptive network based on fuzzy inference system (LDA-ANFIS). They applied the proposed system on hepatitis dataset from UCI repository. By using LDA, the researchers have extracted 8 features from the dataset. They imputed all the missing value in the records. In their experiment 95 and 60 samples were assigned to training and testing dataset respectively. Their proposed ANFIS classifier comprises 5 layers, bell-shaped membership function, and 256 rules. The mean square error (MSE) and number of epochs are 0.001 and 1718, respectively. With the mentioned MSE and epochs, the researchers have achieved accuracy of 94.16%.

Karabatak and Ince [24] developed a breast cancer detection system using association rules (AR) and neural network (NN). The dataset used for this study is taken from UCI. The researchers have used two different techniques of AR, AR1 and AR2, to reduce the features of the dataset. AR1 feature selection eliminates the attributes if it depends on others with threshold value of support and confidence, whereas AR2 selects the frequent attributes in each class. By using AR1 method they reduced the feature size to eight from nine. The AR2 method selects four attributes based on their frequency. In their study, both AR1NN and AR2NN have 11 and 1 neuron in their hidden and output layer respectively. The activation functions selected by the researchers are tangent sigmoid and linear for hidden and output layer, respectively. They have used Levenberg-Marquardt backpropagation learning rule and MSE of 0.01. They have achieved accuracy of 95.6% with AR1 reducts.

Kahramanli and Allahverdi [25] proposed a hybrid neural network system by integrating artificial neural network (ANN) and fuzzy neural network (FNN) to diagnose diabetes and heart disease. The two datasets, namely, Pima Indian diabetes and Cleveland heart disease dataset, are taken from UCI machine learning repository. Missing value for heart disease is imputed by the mean value of the attribute to which the tuple class belongs. The researcher used two separate phases (FNN and ANN1) and one more phase (ANN2) by using the output of the two phases as input. They have used triangular membership function and maximum defuzzifier for FNN phase. Crisp value is taken as input and sigmoid activation function is applied for both hidden and output layers for ANN backpropagation phase. The output of the first two phases becomes input for ANN2. Their proposed system achieved accuracy of 84% and 87% for diabetes and heart disease dataset, respectively.

Compared to the work discussed in the literature, the proposed system is different in the following ways: first, the system handles the missing values of the dataset by rejecting or imputing records. If the quantum of missing value is greater than twenty-five percent, the system rejects the record, else the system imputes by the most frequent value of the attribute belonging to the same class label of the record. After handling the missing value, dimension reduction is performed by using rough set indiscernibility method. Selected reducts are given as input to the artificial neural network for classification.

## 3. System Architecture

**Figure 1:**
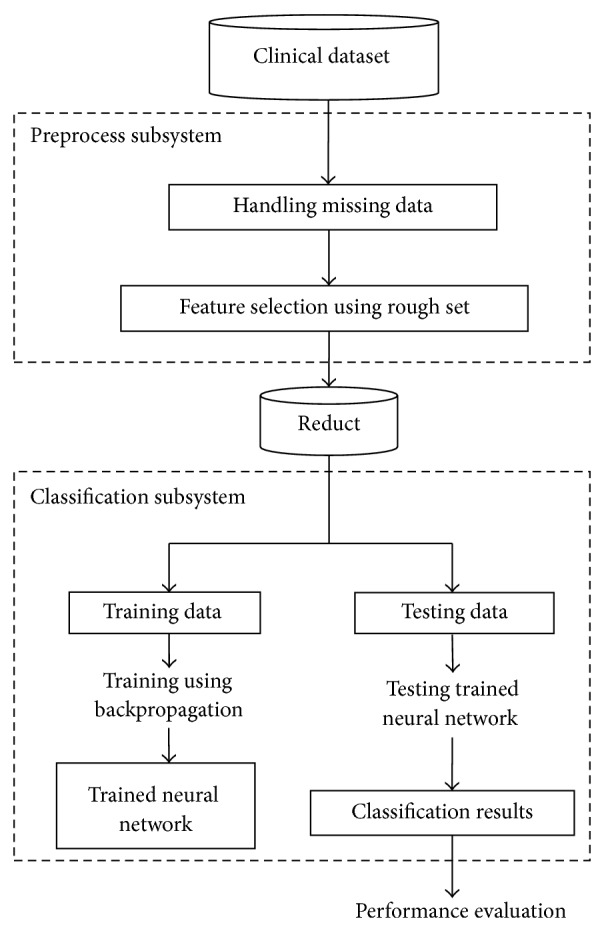
System architecture.

The architecture of the proposed system is shown in Figure 1.

### 3.1. Dataset Description

For this study three clinical datasets have been selected from the UCI machine learning repository. These are hepatitis dataset, Wisconsin breast cancer dataset, and Statlog heart disease dataset. Hepatitis dataset consists of 155 samples with 19 case attributes and a class label. The class label predicts whether the patient with hepatitis will live or not. The dataset has 32 “Die” and 123 “Live” instances.  Table 1 describes the attributes of the hepatitis dataset.

The Wisconsin breast cancer dataset identifies whether the patient's breast tissue is malignant or benign. Benign class has 458 (65.5%) records and malignant class holds 241 (34.5%) records. After excluding the sample code number, the dataset consists of nine features and a class attribute. All attributes have a data type of integer value ranging from 1 to 10. It holds 16 samples with missing value. All the missing values belong to the attribute of bare nucleoli.  Table 2 briefs the attributes of Wisconsin breast cancer dataset.

The third clinical dataset for this study is the Statlog heart disease. This dataset has 270 samples with 13 features and a class attribute. There are no missing values in this dataset. A description of the dataset is given in Table 3.

### 3.2. Handling Missing Value

Handling of missing values is a data preprocessing technique to obtain a smooth dataset. The common methods include ignoring the tuple that holds missing value, imputing with the mean, or imputing with the most frequent value [1]. Hepatitis and breast cancer dataset have missing values but not Statlog heart disease dataset. In this study, missing value is handled as follows. If the percentage of missing value in a tuple is greater than or equal to 25%, then reject that tuple from the dataset or else impute it by the most frequent value of the attribute in the class that belongs to the tuple. The same applies to attributes too.

After handling missing values, the hepatitis dataset is reduced to 147 samples with 18 attributes by rejecting Protime attribute and 8 tuples from the dataset. For breast cancer dataset, all the missing values are imputed by the most frequent value of the attribute of the class label that belongs to the tuple.

### 3.3. Feature Selection Using Rough Set

Feature selection using rough set theory has been widely used in data analysis [9]. The selected subset of attributes, which has the same equivalence relation of entire attribute, is referred to as reduct (Red (R)) [7–9]. In this work, rough set theory indiscernibility relation method is used for feature selection.

*Information System Table*. Information system table is a two-dimensional table (column and row). The row shows definite number of objects, whereas the column shows the attribute value and class label of the objects [9]. It can be presented as *I* = (*U*, *A* ∪ *D*) where *U* is finite set of objects, *A* is set of attribute, and *D* is decision (class label).  Table 4 shows sample of information system table based on three attributes.

*Indiscernibility Relation  *(*IND*(*p*)). It is an equivalence relation. Let *a* ∈ *A*, *P*⊆*A*; indiscernibility relation is defined as

(1) $$\begin{array}{c} \text{IND}\left(P\right)=\left\{\left(x,y\right)\in U\times U:\forall a\in P,a\left(x\right)=a\left(y\right)\right\}. \end{array}$$

Indiscernibility relation can be explained by block partitioning of objects. In Table 4 objects *P*_1_ and *P*_4_ have the same value (2) for attribute Chp, objects *P*_3_, *P*_5_, and *P*_6_ also have the same value (3), but object *P*_2_ has a unique value (1). Based on this IND(Chp) = {{*P*_1_, *P*_4_}, {*P*_2_}, {*P*_3_, *P*_5_, *P*_6_}}.

Upper approximation of a set *X* ($\bar{R}(X)$) includes all objects of information system table which possibly belongs to the class *X*. Lower approximation of set *D* ($\underline{R}(D)$) is the set of objects of the information system table which certainly belongs to the class *X*. The set of all objects that belong to lower approximation is referred to as positive region (Pos_*R*_(*D*)). The difference between upper approximation set and lower approximation set is referred to as boundary region (Bnd*R*(*X*)). Equations (2)–(5) show mathematical formula for $\left(\bar{R}(X)\right)$, $\left(\underline{R}(D)\right)$, (Bnd*R*(*X*)), and (Pos_*R*_(*D*)):

(2) $$\begin{array}{c} \bar{R}\left(D\right)=\left\{x\in U:R\left(X\right)\cap x\neq \emptyset \right\}, \end{array}$$

(3) $$\begin{array}{c} \underline{R}\left(D\right)=\left\{x\in U:R\left(X\right)\subseteq x\right\}, \end{array}$$

(4) $$\begin{array}{c} \text{Bnd}R\left(D\right)=\bar{R}\left(D\right)-\underline{R}\left(D\right), \end{array}$$

(5) $$\begin{array}{c} \text{Po}{\text{s}}_{R}\left(D\right)=\underset{x\in U/\text{IND}(P)}{⋃}\underline{R}\left(D\right). \end{array}$$

The minimal subset of attributes with the same property as that of whole attribute is called reduct (Red (R)). The intersection of the elements of reducts is called core (*C*). By excluding empty set and whole conditional attribute, the total number of minimal subsets of attributes (*S*) competing for reducts becomes

(6) $$\begin{array}{c} S=2^{n}-2, \end{array}$$

where *n* stands for total number of attributes.

The number of minimal subset of attributes generated in each number of attributes (*S*_*i*_) can be computed by rule of combination (*C*). It becomes

(7) $$\begin{array}{c} S_{i}=C_{ni}^{N}, \end{array}$$

where *ni* = number of attributes in minimal subset.

*Feature Selection*. The steps to select the reduct are as follows.

**Step 1 .**

Find upper approximation of each class using (2).

**Step 2 .**

Find lower approximation of each class by applying (3).

**Step 3 .**

Compute positive region of the universe by implementing (5).

**Step 4 .**

Calculate the number of minimal subsets of attributes by using (6) and (7).

**Step 5 .**

Find indiscernibility of each subset of attributes of a positive region using (1).

**Step 6 .**

Compare the indiscernibility of each subset to the indiscernibility of the whole attribute.

**Step 7 .**

Select equivalent indiscernibility as reduct.

Based on the given steps, the reduct of Table 4 is computed as shown in Algorithm 1.

Information system table shown in Table 4 has two reducts as shown in Algorithm 1. They are {Chp, Vessel} and {ECG, Vessel}.

### 3.4. Training and Testing the Classifier

**Figure 2:**
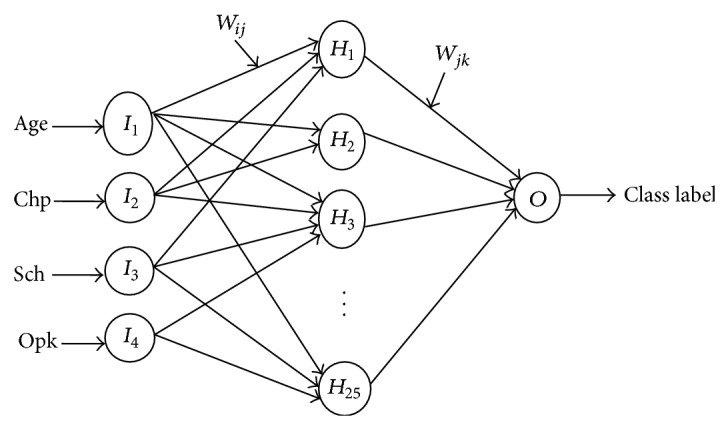
Architecture of BPNN for reduct R1 of Stalog Heart disease dataset.

The experimental analysis of the proposed work has been done by dividing the dataset into training-testing sets in the ratio of (80–20), (70–30), and (60–40). 10-fold cross validation technique is used for validating the training phase of the classifier. The attributes of the selected reducts are connected to the input layer of BPNN. The architecture of the proposed BPNN with minimal set of attributes, pertaining to the Statlog heart disease dataset, is shown in Figure 2.

Besides feature selection, suitable parameters of the proposed system help to improve the performance of the classifier. The parameters of the proposed BPNN to obtain higher performance are indicated in Table 5.

### 3.5. Performance Evaluation

The confusion matrix, shown in Table 6, consists of true positive (TP), false positive (FP), true negative (TN), and false negative (FN). Accuracy, sensitivity, and specificity of rough set backpropagation method are computed based on the confusion matrix. Accuracy is the percentage of the sample data that are correctly classified by the classifier. Sensitivity is the percentage of how many samples are correctly classified as true positive. Specificity is the percentage of how many true negatives are predicted by the classifier. True positive rate (TPR) and false positive rate are also the other performance metrics. TPR is the same as sensitivity; FPR becomes 1 − specificity. Equations (8), (9), (10), (11), and (12) defines the performance metrics:

(8) $$\begin{array}{c} \text{Accuracy}=\frac{\text{TP}+\text{TN}}{\text{TP}+\text{TN}+\text{FP}+\text{FN}}\times 100\%, \end{array}$$

(9) $$\begin{array}{c} \text{Sensitivity}=\frac{\text{TP}}{\text{TP}+\text{FN}}\times 100\%, \end{array}$$

(10) $$\begin{array}{c} \text{Specificity}=\frac{\text{TN}}{\text{TN}+\text{FP}}\times 100\%, \end{array}$$

(11) $$\begin{array}{c} \text{TPR}=\frac{\text{TP}}{\text{TP}+\text{FN}}, \end{array}$$

(12) $$\begin{array}{c} \text{FPR}=\frac{\text{FP}}{\text{FP}+\text{TN}}. \end{array}$$

Besides the above performance metrics, a graphical measuring tool, receiving operating characteristics (ROC), is also used. ROC curve shows the cut-off between TPR and FPR.

## 4. Experimental Results

The experiment on the selected clinical datasets has been performed using MATLAB tool version 7.10, release R2010a.

### 4.1. Feature Selection Result

Hepatitis dataset has a set of 262142 (2^18^ − 2) competing for reduct. Out of these, 562 subsets of attributes fulfill the equivalent indiscernibility relation to be a reduct. Each reduct has 9 to 17 attributes.  Table 7(a) shows the number of hepatitis reducts.

Selected reducts of hepatitis dataset are shown in Table 8. Each attribute is represented by 1 or 0. If the attribute exists in the set, it is represented by “1” or else it becomes “0” based on the following sequence of attributes {age, sex, steroid, antivirals, fatigue, malaise, anorexia, liver big, liver firm, spleen palpable, spiders, ascites, varices, bilirubin, Alk phosphate, Sgot, albumin, and histology}. Two reducts are selected from each of 10 to 13 sets of attributes and one reduct is selected from 9 sets of attributes. More or less all selections are trying to focus on attributes age, steroid, anorexia, fatigue, Alk phosphate, Sgot, albumin, histology, and some other attributes to get more accurate results.

Breast cancer dataset has a total of 510 (2^9^ − 2) sets of attributes competing for reducts. The indiscernibility of all subsets is not fully similar, but partial. For this study we have taken those subsets which have more than 90% similarity to the indiscernibility of all attributes. 11 subsets that fulfill the threshold value are selected as reducts. Nine out of 11 reducts are selected for classification analysis. Five reducts are selected from a subset of 8 attributes and four reducts are from a subset of 7 attributes. All the nine reducts are selected based on the accuracy achieved. The reducts of breast cancer are shown in Table 7(b).  Table 9 shows the selected attribute in each reduct. The sequence of attributes is {clump thickness, uniformity of cell size, uniformity of cell shape, marginal adhesion, single epithelial cell size, bare nuclei, bland chromatin, normal nucleoli, and mitoses}.

Heart disease dataset has a subset of 8190 (2^13^ − 2) sets of attributes competing for reducts. The proposed feature selection method generates 4473 reducts with the size of 3 to 12 attributes in a subset.  Table 7(c) shows the reducts of heart disease dataset. Nine reducts are selected for classification purpose. Two reducts are selected from each candidate reduct with 4 to 7 attribute sets. One more reduct is added from a subset of 6 attributes. More or less the selection of reducts is done by including vessel attributes and excluding sex, Fbs, and slope and adding some other attributes to obtain improved results.  Table 10 describes the attributes of the selected reducts as the sequence of the following: {age, sex, Chp, Bp, Sch, Fbs, Ecg, Mhrt, Exian, Opk, slope, vessel, and Thal}.

### 4.2. Classification Result

**Figure 3:**
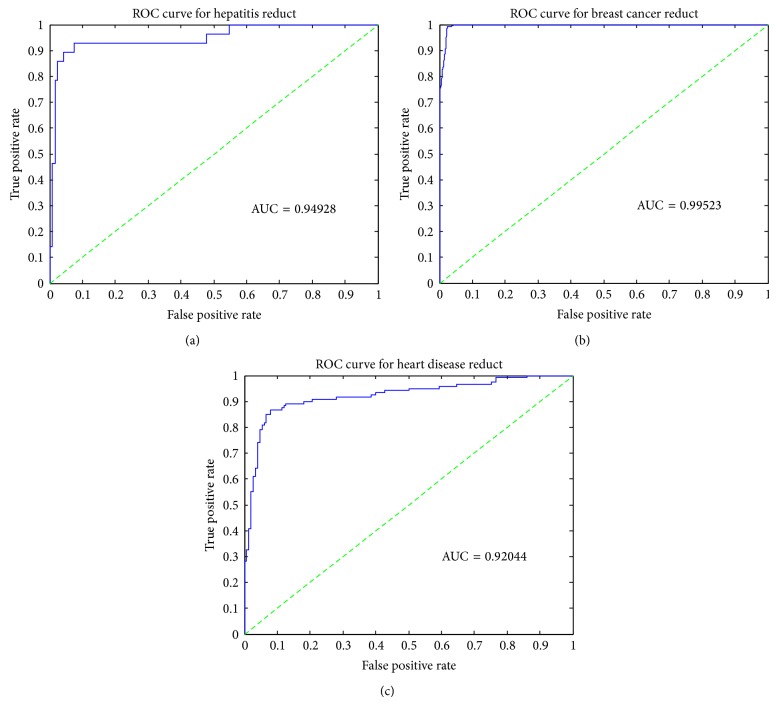
ROC graph for (a) Hepatitis Reduct-R9, (b) Breast cancer Reduct-R1, (c) Heart disease Reduct-R6.

**Figure 4:**
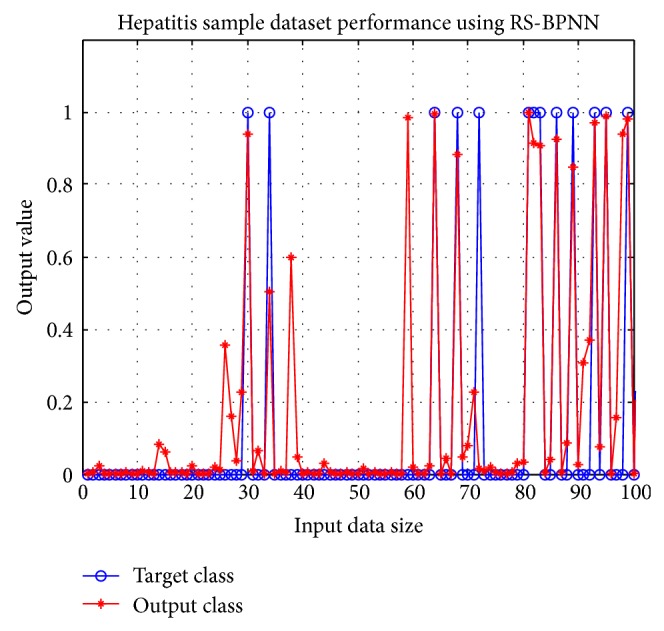
Result of Hepatitis Reduct-R9.

By using suitable parameters for BPNN as shown in Table 5, the classification result of the selected reducts of each dataset is discussed. All the selected hepatitis reducts have achieved accuracy of more than 90%. The best accuracy scored is 97.3% obtained from Reduct-R9 with attribute set of {age, steroid, antivirals, fatigue, liver big, liver firm, spiders, varices, bilirubin, Alk phosphate, Sgot, albumin, and histology} and the data division of (80–20). Accuracy of the selected reducts of hepatitis is given in Table 11(a). The ROC graph of Reduct-R9 is displayed in Figure 3(a). The bold line on the graph shows border region of the ROC, whereas the broken line drawn from the bottom left corner to upper right corner is the ideal line to show that the AUC is greater than 0.5.  Figure 4 shows the performance of target class and output result or Reduct-R9 by taking the first 100 input records.

**Figure 5:**
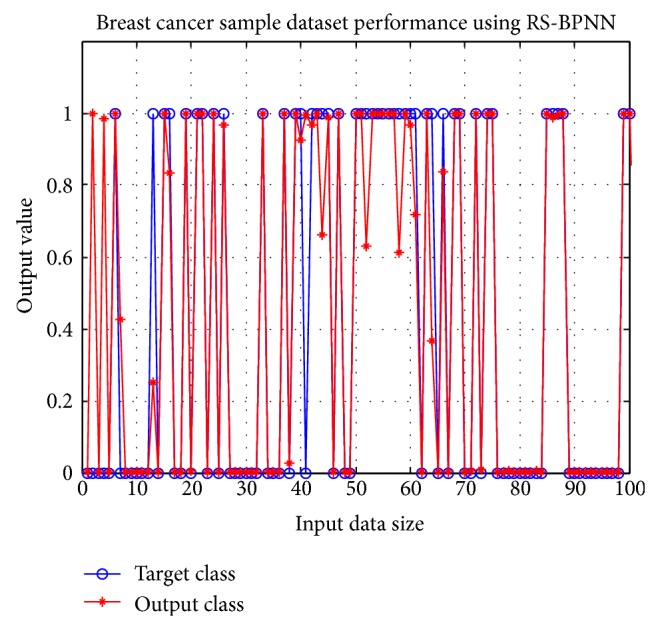
Result of Breast cancer Reduct-R1.

Breast cancer Reduct-R1 with seven features (clump thickness, uniformity of cell size, uniformity of cell shape, marginal adhesion, single epithelial cell size, bare nuclei, and bland chromatin) obtains accuracy of 98.6% with a data division of 80–20. The remaining breast cancer reducts and all features score accuracy greater than 97%. The classification accuracy of breast cancer reducts is mentioned in Table 11(b). The ROC graph of Reduct-R1 is displayed in Figure 3(b). The performance of target class and output result or Reduct-R1 by taking the first 100 input records is shown in Figure 5.

**Figure 6:**
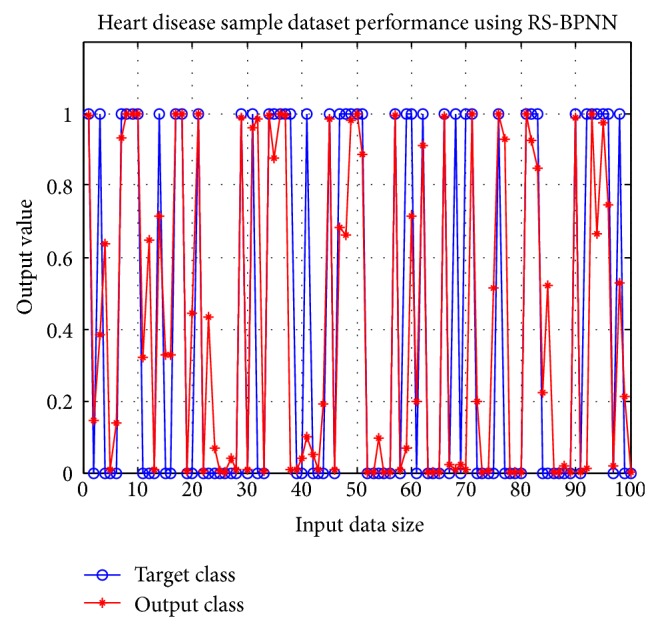
Result of Heart disease Reduct-R6.

The best performance result for heart disease is obtained from Reduct-R6 with 6 attributes (Chp, Sch, Ecg, Opk, vessel, and Thal). It achieves 90.4% accuracy. The other reducts and full attributes score accuracy of 77%. Table 11(c) shows the performance evaluation of heart disease reducts. ROC graph of Reduct-R6 is shown in Figure 3(c).  Figure 6 shows the difference in the obtained results and expected results for 100 records.

Table 12 summarizes the best performed reducts of each dataset with a data division of training-testing of 80–20. It provides major performance metrics of the proposed system.

### 4.3. Performance Comparison

The proposed method, RS-BPNN, provides higher performance when compared to earlier methods proposed by other authors and conventional methods.  Table 13 shows a comparison of the proposed method with other authors' methods.  Table 14 compares the proposed system's experimental result with conventional methods such as CHAID (Chi-squared automatic interaction detection), CRT (classification and regression tree), MLP (multilayer perceptron), and RBFN (radial basis function network).

## 5. Conclusion and Future Work

This paper combines rough set theory with backpropagation neural network to classify the clinical dataset. The proposed algorithm is applied in three clinical datasets from UCI. These are hepatitis, Wisconsin breast cancer, and Statlog heart disease datasets. The rough set theory is used to produce minimal subset of attributes to represent the whole features of the dataset. From rough set theory the proposed method used indiscernibility relation method to select the reducts. From hepatitis dataset indiscernibility relation method produced 562 reducts with the attribute ranging from 9 to 13. From breast cancer disease dataset the proposed method obtained 9 reducts with 7 and 8 attributes. From heart disease dataset the proposed method produced 4473 reducts with attribute of 3 to 12. Nine reducts and all attributes from each of the datasets are used for comparison of classification result. An artificial neural network with backpropagation learning algorithm is used for classification. The proposed work achieves superior performance when compared to recent and conventional works. It is not always true that the reduct with less number of attributes gives highest classification accuracy. In this study hepatitis and heart disease dataset have got their best accuracy performance with the reduct that consists of 13 and 6 attributes, respectively. But there were reducts with less number of attributes in both datasets. Breast cancer dataset obtained its higher performance with a reduct of least number of attributes.

The proposed method of rough set theory examines the set of attributes for indiscernibility relation method to obtain reducts. In future, the use of hybrid methods of rough set theory with optimization techniques like particle swarm optimization (PSO), ant colony optimization (ACO), genetic algorithm (GA), and bacterial foraging (BF) can be experimented with many more datasets.

## Figures and Tables

**Algorithm 1:**
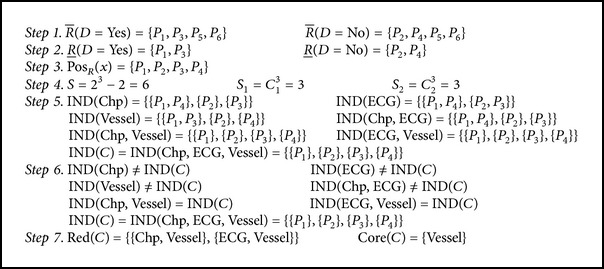
Steps to extract reduct from dataset.

**Table 1:** Description of hepatitis dataset.

Number	Attribute name	Domain values	Number of missing values
1	Age	10, 20, 30, 40, 50, 60, 70, 80	0
2	Sex	Male, female	0
3	Steroid	No, yes	1
4	Antivirals	No, yes	0
5	Fatigue	No, yes	1
6	Malaise	No, yes	1
7	Anorexia	No, yes	1
8	Liver big	No, yes	10
9	Liver firm	No, yes	11
10	Spleen palpable	No, yes	5
11	Spiders	No, yes	5
12	Ascites	No, yes	5
13	Varices	No, yes	5
14	Bilirubin	0.39, 0.80, 1.20, 2.00, 3.00, 4.00	6
15	Alk phosphate	33, 80, 120, 160, 200, 250	29
16	Sgot	13, 100, 200, 300, 400, 500	4
17	Albumin	2.1, 3.0, 3.8, 4.5, 5.0, 6.0	16
18	Protime	10, 20, 30, 40, 50, 60, 70, 80, 90	67
19	Histology	No, yes	0
20	Class	Die, live	0

**Table 2:** Description of breast cancer dataset.

Number	Attribute name	Domain	Missing value
1	Clump thickness	1–10	0
2	Uniformity of cell size	1–10	0
3	Uniformity of cell shape	1–10	0
4	Marginal adhesion	1–10	0
5	Single epithelial cell size	1–10	16
6	Bare nucleoli	1–10	0
7	Bland chromatin	1–10	0
8	Normal nucleoli	1–10	0
9	Mitosis	1–10	0
10	Class	2 for benign, 4 for malignant	0

**Table 3:** Attribute information of Statlog heart disease dataset.

Number	Attribute	Description	Data type	Domain
1	Age	Patient age in year	Numerical	29 to 77

2	Sex	Gender	Binary	0 = female 1 = male

3	Chp	Chest pain type	Nominal	1 = typical angina, 2 = atypical angina 3 = nonanginal pain, 4 = asymptomatic

4	Bp	Resting blood pressure	Numerical	94 to 200

5	Sch	Serum cholesterol	Numerical	126 to 564

6	Fbs	Fasting blood sugar >120 mg/dL	Binary	0 = false 1 = True

7	Ecg	Resting electrocardiographic result	Nominal	0 = normal 1 = having ST-T wave abnormality 2 = left ventricular hypertrophy

8	Mhrt	Maximum heart rate	Numerical	71 to 200

9	Exian	Exercise induced angina	Binary	0 = no 1 = yes

10	Opk	Old peak	Numerical	Continuous (0 to 6.2)

11	Slope	Slope of peak exercise ST segment	Nominal	1 = upsloping 2 = flat 3 = downsloping

12	Vessel	Number of major vessels	Nominal	0 to 3

13	Thal	Defect type	Nominal	3 = normal, 6 = fixed defect, 7 = reversible defect

14	Class	Heart disease	Binary	0 = absence, 1 = presence

**Table 4:** Sample of information system table of heart disease.

*U*	*A*			*D*
	Chp	ECG	Vessel	Class
*P*_1_	2	0	3	Yes
*P*_2_	1	2	1	No
*P*_3_	3	2	3	Yes
*P*_4_	2	0	0	No
*P*_5_	3	0	0	No
*P*_6_	3	0	0	Yes

**Table 5:** Parameters of BPNN.

Number of layers	Input layer: 1 with 9–13 features for hepatitis 9 features for breast cancer 3–7 features for heart disease
	Hidden layer: 1 with 25 nodes (*H*_1_, *H*_2_,…, *H*_25_)
	Output layer: 1 with class label (0 or 1)

Activation function	Hidden layer: tangent sigmoid
	Output layer: linear

Learning algorithm	Backpropagation

Dataset division	Random division

**Table 6:** Confusion matrix.

		Predicted class	
		Positive	Negative
Actual class	Positive	TP	FN
	Negative	FP	TN

**Table:** (a) Reduct for hepatitis dataset

Number of attributes	9	10	11	12	13	14	15	16	17
Number of subsets	48620	43758	31824	18564	8568	3060	816	153	18
Number of reducts	2	19	71	137	142	104	43	43	1

**Table:** (b) Reduct for breast cancer dataset

Number of attributes	1	2	3	4	5	6	7	8
Number of subsets	9	36	84	126	126	84	36	9
Selected reducts	—	—	—	—	—	—	5	6

**Table:** (c) Reduct for heart disease dataset

Number of attributes	3	4	5	6	7	8	9	10	11	12
Number of subsets	286	715	1287	1716	1716	1287	715	286	78	13
Number of reducts	9	90	365	811	1127	1043	657	280	78	13

**Table 8:** Selected reducts for hepatitis dataset.

Reduct	Number of attributes	Attribute set
*R*1	9	[1 0 1 0 1 0 0 1 1 0 0 0 0 1 1 1 1 0]
*R*2	10	[1 1 1 0 1 0 0 1 1 0 0 0 0 1 1 1 1 0]
*R*3	10	[1 0 1 0 1 0 0 1 0 1 1 0 0 1 1 1 1 0]
*R*4	11	[1 0 1 0 1 1 0 1 1 0 1 0 0 1 1 1 1 0]
*R*5	11	[1 0 1 0 1 1 0 1 1 1 0 0 0 1 1 1 1 0]
*R*6	12	[1 0 1 0 1 0 1 1 0 0 1 1 1 1 1 1 1 0]
*R*7	12	[1 0 1 0 1 0 1 1 0 1 1 0 1 1 1 1 1 0]
*R*8	13	[1 1 1 1 1 0 0 0 0 1 1 1 0 1 1 1 1 1]
*R*9	13	[1 0 1 1 1 0 0 1 1 0 1 0 1 1 1 1 1 1]
All	18	[1 1 1 1 1 1 1 1 1 1 1 1 1 1 1 1 1 1]

**Table 9:** Example of reducts of breast cancer dataset.

Reduct	Attribute size	Attribute members
*R*1	7	[1 1 1 1 1 1 1 0 0]
*R*2	7	[1 1 1 1 1 0 1 1 0]
*R*3	7	[1 1 1 0 1 1 1 1 0]
*R*4	7	[1 0 1 1 1 1 1 1 0]
*R*5	8	[1 1 1 1 1 1 1 1 0]
*R*6	8	[1 1 1 1 1 1 1 0 1]
*R*7	8	[1 1 1 1 1 0 1 1 1]
*R*8	8	[1 1 1 0 1 1 1 1 1]
*R*9	8	[1 0 1 1 1 1 1 1 1]
All	9	[1 1 1 1 1 1 1 1 1]

**Table 10:** Selected reducts for heart disease dataset.

Reduct	Attribute size	Attribute set
*R*1	4	[1 0 1 0 1 0 0 0 0 1 0 0 0]
*R*2	4	[1 0 1 0 0 0 0 1 0 0 0 1 0]
*R*3	5	[1 0 1 0 0 0 0 1 0 1 0 1 0]
*R*4	5	[1 0 1 1 0 0 0 1 0 0 0 1 0]
*R*5	6	[1 0 1 1 0 0 0 0 1 0 0 1 1]
*R*6	6	[0 0 1 0 1 0 1 0 0 1 0 1 1]
*R*7	6	[0 0 1 0 1 0 0 1 0 1 0 1 1]
*R*8	7	[0 1 0 1 1 0 1 0 0 0 1 1 1]
*R*9	7	[0 0 0 0 1 1 0 1 1 1 0 1 1]
All	13	[1 1 1 1 1 1 1 1 1 1 1 1 1]

**Table:** (a) Hepatitis accuracy (%)

Reduct	(80–20)	(70–30)	(60–40)
*R*1	95.2	93.9	91.2
*R*2	96.6	92.5	93.2
*R*3	93.9	91.2	92.5
*R*4	95.2	91.8	91.2
*R*5	93.9	91.8	94.6
*R*6	95.9	94.6	91.8
*R*7	95.2	93.2	91.2
*R*8	95.2	92.5	91.8
*R*9	97.3	95.9	93.9
All features	87.8	87.1	86.4

**Table:** (b) Breast cancer accuracy (%)

Reduct	(80–20)	(70–30)	(60–40)
*R*1	98.6	98.3	97.6
*R*2	97.6	97.4	97.4
*R*3	98.4	98.0	97.6
*R*4	98.4	98.1	97.4
*R*5	98.0	97.9	97.6
*R*6	98.0	98.0	97.9
*R*7	97.9	97.7	97.1
*R*8	98.4	98.0	97.3
*R*9	98.0	97.7	97.6
All features	97.7	97.7	97.4

**Table:** (c) Heart Disease Accuracy

Reduct	(80–20)	(70–30)	(60–40)
*R*1	79.6	78.1	77.4
*R*2	81.1	81.1	80.4
*R*3	84.8	84.8	84.4
*R*4	85.9	83.0	83.3
*R*5	88.9	84.8	84.1
*R*6	90.4	88.1	87.8
*R*7	88.1	87.0	85.9
*R*8	86.3	85.2	83.3
*R*9	88.5	85.6	84.8
All features	84.4	84.1	84.8

**Table 12:** Performance measure of best reducts of each dataset.

Dataset	Reduct	TP	FN	TN	FP	Accuracy (%)	Sensitivity (%)	Specificity (%)	TPR	FPR	AUC
Hepatitis	Reduct-*R*9	26	2	117	2	97.30	98.32	97.28	0.93	0.017	0.9492
Breast cancer	Reduct-*R*1	238	3	451	7	98.60	98.76	98.57	0.99	0.015	0.9952
Heart disease	Reduct-*R*7	102	18	142	8	90.40	94.67	90.37	0.85	0.053	0.9204

**Table 13:** Comparison of proposed system with recent works.

Author	Technique	Accuracy of dataset (%)		
		Hepatitis	Breast cancer	Heart disease
Sartakhti et al. (2012) [16]	SVM-SA	96.25	—	—
Chen et al. (2011) [20]	LFDA-SVM	96.77	—	—
Çalişir and Dogantekin (2011) [21]	PCA-LSSVM	96.12	—	—
Bascil and Temurtas (2011) [26]	MLNN	91.87	—	—
Dogantekin et al. (2009) [23]	LDA-ANFIS	94.16	—	—
Polat and Güneş (2006) [27]	FS_AIRS	94.12		
Zheng et al. (2014) [28]	K-SVM	—	97.38	—
Karabatak and Ince (2009) [24]	AR_NN	—	95.60	—
Shao et al. (2014) [14]	MARS-LR	—	—	83.93
Anooj (2012) [17]	Weighted fuzzy	—	—	62
Vijaya et al. (2010) [22]	Fuzzy neurogenetic	—	—	80
Kahramanli and Allahverdi (2008) [25]	ANN-FNN	—	—	87
Proposed method	RS-BPNN	97.3	98.6	90.4

**Table 14:** Comparison of proposed system with conventional methods.

Technique	Accuracy (%)		
	Hepatitis	Breast cancer	Heart disease
CHAID	80.8	92.7	76.6
CRT	79.4	92.4	76.6
MLP	89.0	96.1	83.3
RBFN	84.6	94.0	84.6
RS-BPNN	97.3	98.6	90.4
